# Supplementary material for: Mesenchymal stem cells transfer mitochondria to allogeneic Tregs in an HLA-dependent manner improving their immunosuppressive activity
Source: Nat Commun. 2022 Feb 14;13:856. doi: 10.1038/s41467-022-28338-0 (PMC8844425; doi:10.1038/s41467-022-28338-0)
Supplement: Supplementary file 1 — Supplementary Information [file 41467_2022_28338_MOESM1_ESM.pdf]

Supplementary Information

Supplementary Figures

Supplementary Figure 1 Contact with allogeneic ASCs increases expression of CD69 in Tregs

Supplementary Figure 2 Gating strategy for analysis of proliferation suppression assay

Supplementary Figure 3 Gating strategy for analysis of ASC derived organelle transfer

Supplementary Figure 4 Frequency of active mitochondria in ASCs is decreased after direct coculture with allogenic Tregs

Supplementary Tables

Supplementary Table 1 High resolution typing of HLA of Treg and ASC donors

Supplementary Table 2 Increased release of proinflammatory mediators in Treg cocultures with allogenic ASCs correlates with HLA eplet mismatch load

Supplementary Table 3 Correlations between cytokine levels in the cocultures and Treg proliferation

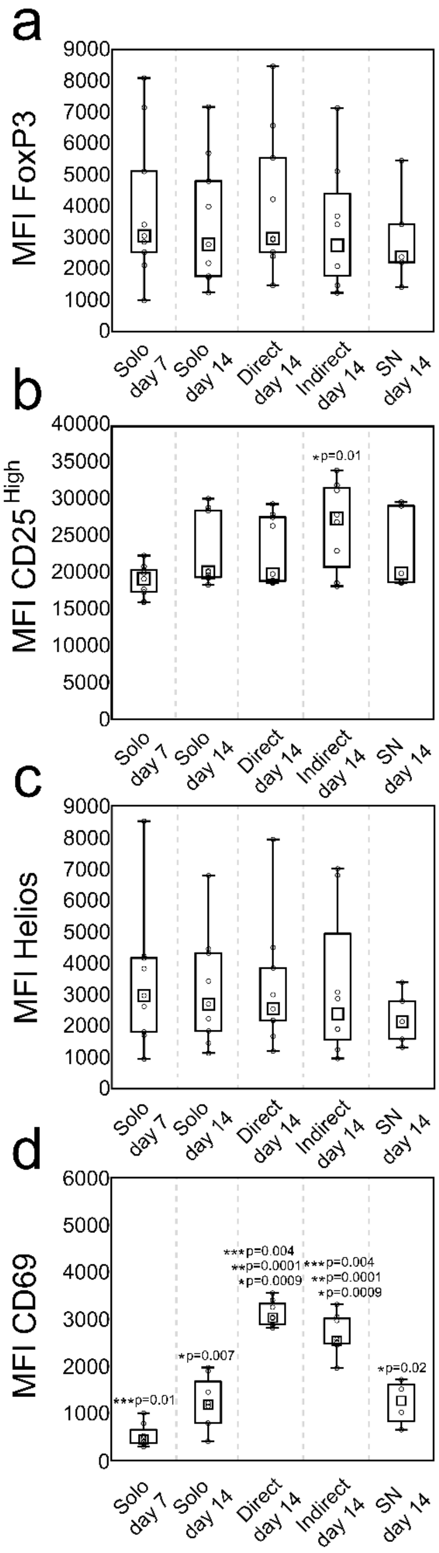

a
MFI FoxP3
9000
8000
7000
6000
5000
4000
3000
2000
1000
0
Solo day 7
Solo day 14
Direct day 14
Indirect day 14
SN day 14
b
MFI CD25 High
40000
35000
30000
25000
20000
15000
10000
5000
0
*p=0.01
Solo day 7
Solo day 14
Direct day 14
Indirect day 14
SN day 14
c
MFI Helios
9000
8000
7000
6000
5000
4000
3000
2000
1000
0
Solo day 7
Solo day 14
Direct day 14
Indirect day 14
SN day 14
d
MFI CD69
6000
5000
4000
3000
2000
1000
0
***p=0.01
*p=0.007
***p=0.004
**p=0.0001
*p=0.0009
***p=0.004
**p=0.0001
*p=0.0009
*p=0.02
Solo day 7
Solo day 14
Direct day 14
Indirect day 14
SN day 14

Supplementary Figure 1 Contact with allogeneic ASCs increases expression of CD69 in Tregs. The figure depicts intensity of (**a**) FoxP3, (**b**) CD25, (**c**) Helios and (**d**) CD69 expression in Tregs positive for the particular marker measured as median fluorescence intensity (MFI). The data are shown for Tregs in monocultures at day 7 and 14 after isolation (Solo day 7 and Solo day 14, respectively), from direct and indirect cocultures at day 14 after Treg isolation that corresponds with day 7 of the coculture (Direct day 14 and Indirect day 14, respectively), and from Treg monocultures supplemented with ASC culture supernatants (SNs) at day 14 after isolation (corresponds with day 7 of culture supplementation with SNs; SN day 14). * defines $p<0.05$ for comparisons between the given condition vs Solo day 7. ** defines $p<0.05$ for differences between the given condition vs Solo day 14. *** defines $p<0.05$ for differences between the given condition vs SN day 14. The data were calculated for 9 independent experiments with a two-sided Mann-Whitney U test with correction. In all boxplots the median is indicated by the symbol within the box, lower and upper bounds of the boxes correspond with the 25th and 75th percentiles. The lower and upper whiskers indicate minimum and maximum values, respectively. Source data are provided as a Source Data file.

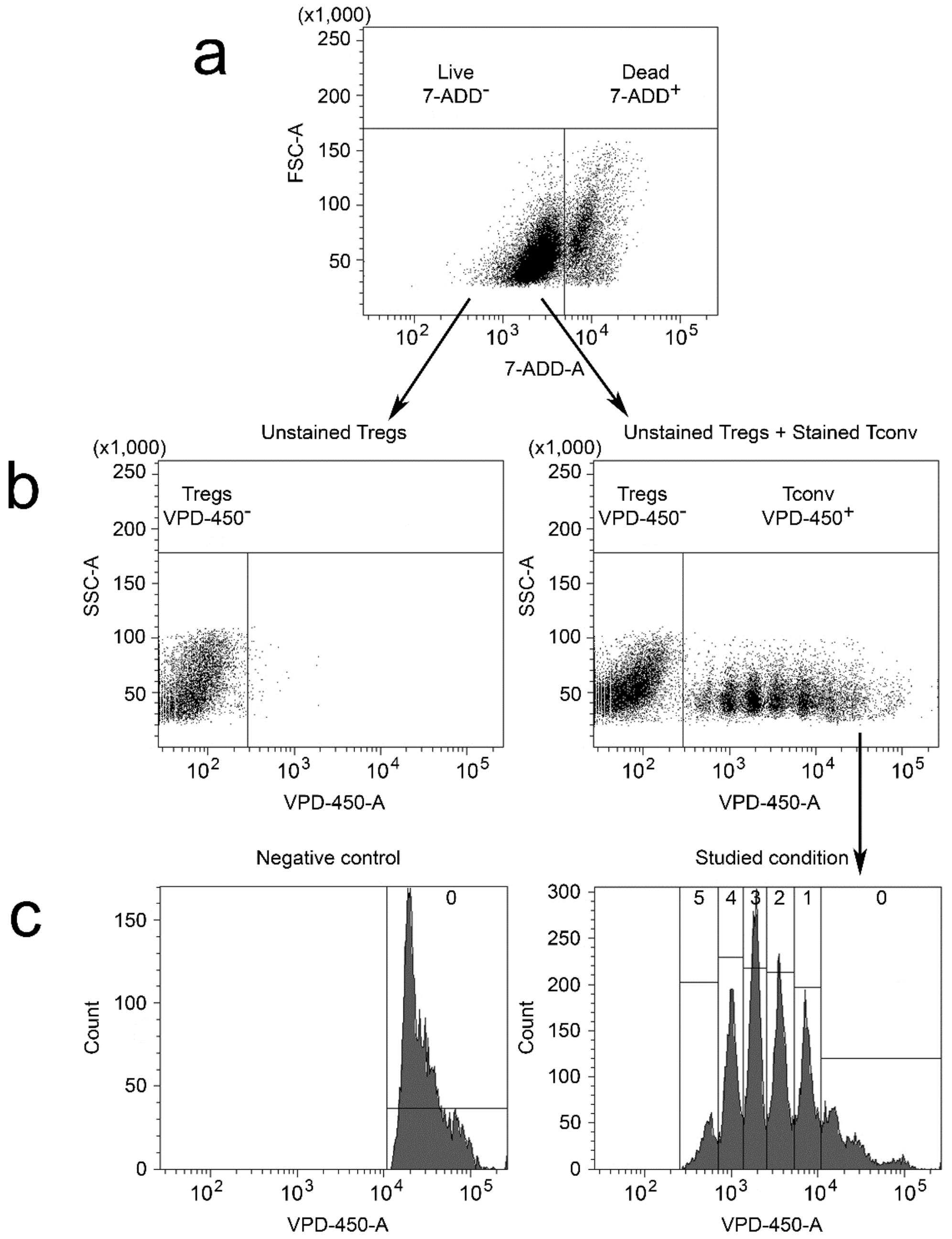

Supplementary Figure 2 Gating strategy for analysis of proliferation suppression assay. (**a**) Before the analysis cells were labelled with 7-ADD in aim to distinguish live (Live 7-ADD$^-$) and dead (Dead 7-ADD$^+$) cells. (**b**) Then, live cells were gated for VPD-450 stained Tconvs (Tconv VPD-450$^+$) and unlabelled Tregs (Tregs VPD450$^-$). Left dot-plot shows autofluorescence of

control unstained Tregs. The control was used in aim to cut off labelled Tconvs from unlabelled Tregs. The right dot-plot depicts an exemplary result of the test. Unstained Tregs (Tregs VPD450^{-}) and VPD-450 labelled Tconvs (Tconv VPD-450^{+}) can be distinguished. Tconvs have divided several times and thus the dye dilution is visible. (**c**) In the next step 7-ADD^{-}VPD-450^{+} Tconvs were analysed. Left histogram shows fluorescence of VPD-450 labelled and unstimulated Tconvs (Negative control). Signal from negative control was used to cut-off non-proliferating cells (0) from division peaks. Right histogram shows an exemplary result of the test. The division peaks are numbered 1 through 5. Proliferation index have been calculated by using the number of cells measured in each division peak.

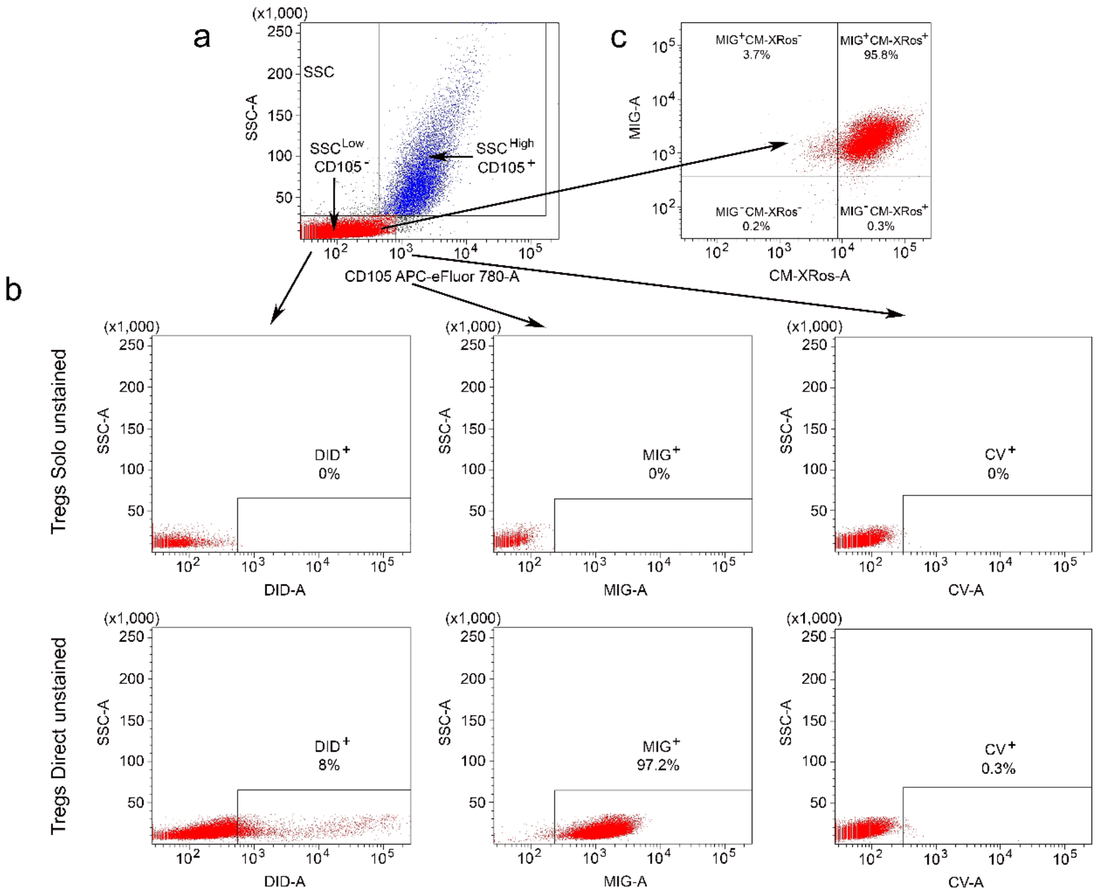

Supplementary Figure 3 Gating strategy for analysis of ASC derived organelle transfer. (a) Tregs and ASCs from the cocultures were distinguished according to different expression of CD105 molecule and SSC (side scatter) values. Tregs and ASCs were identified as $SSC^{Low}CD105^{-}$ and $SSC^{High}CD105^{+}$ cells, respectively. (**b**) Then Tregs were plotted on separate graphs for analysis of uptake of plasmalemma (left panel; plasmalemma uptake visible as increase in DID fluorescence), mitochondria (middle panel; mitochondria uptake visible as increase in MIG fluorescence) and cytosol (right panel; cytosol uptake visible as increase in CV fluorescence). For uptake of each studied cellular element an exemplary result for control unstained Tregs from monocultures (Tregs Solo unstained) and Tregs derived from direct cocultures with labelled ASCs (Tregs Direct unstained) is shown. (**c**) Gating Tregs for analysis of uptake of active (MIG^{+}CM-$XRos^{+}$) and inactive (MIG^{+}CM-$XRos^{-}$) mitochondria derived from ASCs.

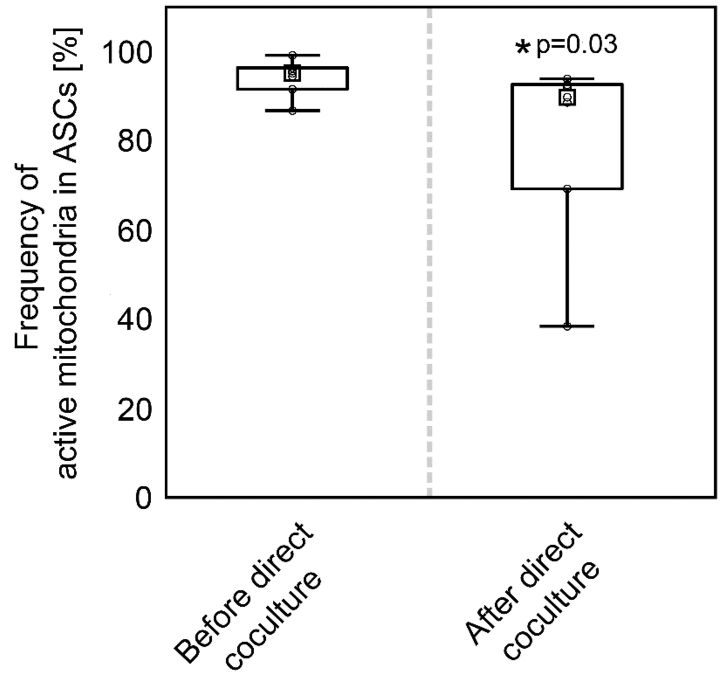

Supplementary Figure 4 Frequency of active mitochondria in ASCs is decreased after direct coculture with allogenic Tregs. The figure depicts frequency of active mitochondria per total mitochondria mass in ASCs before (Before direct coculture) and after (After direct coculture) the direct coculture with Tregs. The data were calculated for 7 independent experiments with a two-sided Mann- Whitney U test with correction. * defines $p<0.05$. In all boxplots the median is indicated by the symbol within the box, lower and upper bounds of the boxes correspond with the 25th and 75th percentiles. The lower and upper whiskers indicate minimum and maximum values, respectively. Source data are provided as a Source Data file.

Supplementary Table 1 High resolution typing of HLA of Treg and ASC donors. The table lists HLA alleles typed with NGS method for all Treg and ASC donors. The data are grouped in pairs that correspond with Treg-ASC cocultures performed.

No. of the Treg-ASC pair	Tregs and ASCs used in the culture	HLA-A*	HLA-A*	HLA-B*	HLA-B*	HLA-C*	HLA-C*	HLA-DRB1*	HLA-DRB1*	HLA-DQB1*	HLA-DQB1*
1.	Tregs 1	03:01:01	26:01:01	07:02:01	35:03:01	04:01:01	07:02:01	12:01:01	15:01:01	03:01:01	06:02:01
	ASCs 1	02:01:01	03:01:01	38:01:01	41:01:01	12:03:01	17:01:01	03:01:01	13:01:01	02:01:01	06:03:01
2.	Tregs 2	02:01:01	26:01:01	07:02:01	51:01:01	01:02:01	07:02:01	11:03:01	15:01:01	03:01:01	06:02:01
	ASCs 2	26:01:01	32:01:01	38:01:01	51:01:01	02:02:02	12:03:01	03:01:01	13:01:01	02:01:01	06:03:01
3.	Tregs 3	01:01:01	02:01:01	15:01:01	57:01:01	03:04:01	06:02:01	04:01:01	13:03:01	03:01:01	03:02:01
	ASCs 3	02:01:01	03:01:01	39:01:01	44:02:01	05:01:01	07:02:01	01:01:01	11:01:01	03:01:01	05:01:01
4.	Tregs 3	01:01:01	02:01:01	15:01:01	57:01:01	03:04:01	06:02:01	04:01:01	13:03:01	03:01:01	03:02:01
	ASCs 4	02:01:01	24:02:01	07:02:01	39:01:01	07:02:01	12:03:01	04:01:01	11:01:01	03:01:01	03:02:01
5.	Tregs 4	02:01:01		27:02:01	51:01:01	02:02:02	15:02:01	11:01:01		03:01:01	
	ASCs 5	02:01:01	11:01:01	27:05:02	39:01:01	02:02:02		13:01:01		06:03:01	
6.	Tregs 5	03:01:01	24:02:01	15:01:01	40:01:02	03:03:01	07:02:01	03:01:01	07:01:01	02:01:01	02:02:01
	ASCs 6	02:01:01	11:01:01	27:02:01	52:01:01	02:02:02	12:02:02	01:01:01	16:01:01	05:01:01	05:02:01
7.	Tregs 6	01:01:01	02:01:01	08:01:01	13:02:01	06:02:01	07:01:01	03:01:01	07:01:01	02:01:01	02:02:01
	ASCs 6	02:01:01	11:01:01	27:02:01	52:01:01	02:02:02	12:02:02	01:01:01	16:01:01	05:01:01	05:02:01
8.	Tregs 7	02:01:01	31:01:02	08:01:01	27:05:02	02:02:02	07:01:01	03:01:01	09:01:02	02:01:01	03:03:02
	ASCs 7	02:01:01	24:02:01	40:02:01		02:02:02		11:01:01		03:01:01	
9.	Tregs 8	03:01:01	30:02:01	18:01:01	56:01:01	01:02:01	05:01:01	01:01:01	03:01:01	02:01:01	05:01:01
	ASCs 8	02:01:01	29:02:01	13:02:01	44:03:01	06:02:01	16:01:01	07:01:01	07:01:01	02:02:01	

Supplementary Table 2 Increased release of proinflammatory mediators in Treg cocultures with allogenic ASCs correlates with HLA eplet mismatch load. The table shows levels of 50 cytokines measured in supernatants collected after 24h and 48h from Treg monocultures (Tregs Solo, n=6), ASC monocultures (ASCs Solo, n=6), direct and indirect Treg-ASC cocultures (Direct, n=6 and Indirect, n=6, respectively). For each cytokine the median values and range in parentheses are given. The differences in cytokine levels between the cultures were calculated with two-sided Mann-Whitney U tests with correction. * defines $p<0.05$ for differences between Direct 24h vs Tregs Solo 24h and Direct 48h vs Tregs Solo 48h. § defines $p<0.05$ for differences between Direct 24h vs ASCs Solo 24h and Direct 48h vs ASCs Solo 48h. # defines $p<0.05$ for differences between Indirect 24h vs Tregs Solo 24h and Indirect 48h vs Tregs Solo 48h. ¤ defines $p<0.05$ for differences between Indirect 24h vs ASCs Solo 24h and Indirect 48h vs ASCs Solo 48h. ** defines $p<0.05$ for differences between Tregs Solo 24h vs ASCs Solo 24h and Tregs Solo 48h vs ASCs Solo 48h. € defines $p<0.05$ for differences between Direct 24h vs Indirect 24h and Direct 48h vs Indirect 48h. Correlations between the cytokine levels and HLA eplet mismatch load for direct and indirect cocultures together (n=12) are listed for HLA-A, -B, -C, -DRB1 and -DQB1 alleles and were analyzed with Spearman's rank correlation. R and p values are given for each correlation. Statistically significant correlations are marked in red. Levels of IL-2 shown in the table resulted from IL-2 supplementation of all the cultures and its consumption by the cells. Thus, IL-2 levels are given only to show various IL-2 milieu in the cultures, but were excluded from analyses.

Cytokine	Tregs Solo		ASCs Solo		Direct		Indirect		Cytokine vs HLA class I mismatch eplet load			Cytokine vs HLA class II mismatch eplet load	
	24h	48h	24h	48h	24h	48h	24h	48h	HLA-A (R/p)	HLA-B (R/p)	HLA-C (R/p)	HLA-DRB1 (R/p)	HLA-DQB1 (R/p)
IL-21	0.00	0.00	0.00	0.00	0.00	0.00	0.00	0.00 (0.00-233.16)	N/A	N/A	N/A	N/A	N/A
IL-35	11.97 (0.00-28.21)**	32.79 (0.00-39.05) **	1049.00 (838.56-1049)	≥1049	≥1049*	≥1049*	≥1049#	≥1049#	N/A	N/A	N/A	N/A	N/A
sCD40L	0.00	0.00 (0.00-6.58)	0.00	0.00	4.38 (0.00-6.88)	8.72 (0.00-12.76)* §	5.43 (0.00-8.81)	7.27 (0.00-8.59)	0.00/ 1.00	0.00/ 1.00	-0.14/ 0.72	0.76/ 0.02	0.00/ 1.00
EGF	0.89 (0.71-1.15)	1.10 (0.95-1.37)	0.95 (0.83-1.23)	0.98 (0.83-1.12)	2.01 (1.64-2.80)*§ €	2.72 (2.45-2.75)*§ €	1.48 (1.28-1.96) #¤	1.87 (1.60-2.29)#¤	0.22/ 0.63	0.29/ 0.52	0.00/ 1.00	0.15/ 0.73	-0.22/ 0.63
Eotaxin	1.46 (1.23-1.84)	1.98 (1.73-2.47)	1.58 (1.30-1.84)	1.68 (1.43-2.06)	14.76 (2.70-28.15)* § €	22.81 (3.17-37.12)* § €	2.27 (1.78-3.87) #¤	2.68 (2.27-8.22)#¤	0.22/0.63	0.55/ 0.20	0.03/ 0.93	0.15/ 0.73	-0.22/ 0.63
FGF-2	0.61 (0.56-0.71) **	0.74 (0.61-0.78)	1.19 (0.67-3.79)	0.72 (0.64-1.13)	1.67 (0.97-4.14)*	1.66 (1.00-3.37)*§	1.19 (0.80-1.49)#	1.15 (0.84-1.46)#¤	-0.01/ 0.96	0.27/ 0.55	0.05/ 0.90	0.79/ 0.03	0.01/ 0.96

FLT-3L	3.24 (2.82-3.55) **	6.00 (0.55-6.81) **	0.56 (0.48-0.62)	0.55 (0.53-0.58)	7.05 (6.26-8.83)*§ €	12.29 (10.06-15.15)* §€	4.32 (3.12-5.23)#¤	8.28 (6.26-11.1)#¤	-0.34/ 0.40	-0.04/ 0.90	0.24/ 0.56	0.12/ 0.76	0.34/ 0.40
Fractalkine	0.59 (0.00-0.97)	0.72 (0.65-0.97)	0.69 (0.00-0.84)	0.75 (0.65-1.00)	1.55 (1.09-1.85)*§ €	1.84 (1.48-3.00)*§ €	1.06 (0.84-1.26)#¤	1.22 (0.97-1.74)#¤	-0.24/ 0.55	0.34/ 0.40	0.36/ 0.36	0.31/ 0.44	0.24/ 0.55
G-CSF	0.50 (0.34-0.73)	0.67 (0.39-0.98)	0.76 (0.50-0.85)	0.98 (0.55-2.69)	126.21 (6.53-377.37) *§	1721.64 (42.66-3469.00) *§	18.89 (1.38-95.37) #¤	274.07 (2.54-512.29)# ¤	0.09/ 0.81	0.68/ 0.06	0.19/ 0.64	0.75/ 0.03	-0.09/ 0.81
GM-CSF	18.28 (15.47-34.26) **	26.40 (1.21-56.60) **	0.00 (0.00-0.99)	0.43 (0.00-1.21)	21.61 (16.14-34.00) §	72.26 (22.18-102.47) §	22.93 (11.15-30.85) ¤	41.43 (20.97-64.46)¤	0.19/ 0.64	0.58/ 0.12	0.04/ 0.90	0.75/ 0.03	-0.19/ 0.64
GROα	0.35 (0.25-1.66) **	0.43 (0.35-18.97) **	7.44 (2.82-27.96)	18.17 (4.16-74.52)	109.63 (43.62-639.45) *§	898.85 (123.88-2041) *§	32.15 (13.01-390.98)# ¤	312.40 (38.20-1015)#¤	0.29/ 0.48	0.68/ 0.06	0.04/ 0.90	0.62/ 0.09	-0.29/ 0.48
IFNα2	1.37 (1.03-1.69)	1.45 (1.01-1.91)	1.15 (0.81-1.43)	1.23 (1.01-1.32)	2.08 (1.03-3.53)§	2.03 (1.69-3.90) *§	1.65 (1.60-2.29) #¤	1.91 (1.73-2.64) # ¤	0.09/ 0.81	-0.09/ 0.81	-0.29/ 0.48	0.75/ 0.03	-0.09/ 0.81
IFNγ	61.02 (14.76-134.43) **	91.45 (1.78-217.73) **	1.34 (1.150-1.74)	1.40 (1.240-1.78)	38.99 (12.80-79.62)§	108.47 (15.48-160.92) §	69.38 (15.40-81.99) ¤	120.50 (13.82-215.92)¤	0.58/ 0.12	0.19/ 0.64	-0.53/ 0.17	0.75/ 0.03	-0.58/ 0.12
IL-1α	0.15 (0.00-1.08)	0.33 (0.00-1.38)	0.00	0.00	0.39 (0.00-0.65)§	0.67 (0.32-1.41) §	0.36 (0.00-0.51)	0.38 (0.00-0.69)¤	0.29/ 0.48	0.48/ 0.21	-0.09/ 0.81	0.75/ 0.03	-0.29/ 0.48

IL-1β	1.71 (1.61-2.40)	2.28 (1.80-2.97) **	1.59 (1.420-2.10)	1.74 (1.470-2.25)	3.93 (2.97-5.74) *§€	6.42 (3.43-8.80) *§€	2.92 (2.00-3.85) #¤	3.18 (2.51-4.06)#¤	0.19/ 0.64	0.19/ 0.64	-0.14/ 0.72	0.50/ 0.20	-0.19/ 0.64
IL-1RA	1.70 (1.60-2.16)	1.95 (1.63-2.84)	1.66 (1.560-1.78)	1.80 (1.700-1.96)	3.29 (2.34-6.07) *§€	4.91 (3.50-12.68) *§	2.28 (1.76-2.84) #¤	3.55 (2.18-5.03)#¤	-0.09/ 0.81	0.29/ 0.48	0.14/ 0.72	0.62/ 0.09	0.09/ 0.81
IL-2	11719.5 (10848-13148)	11615.5 (11112-12728)	12059 (9157-12795)	12101.5 (9280-12638)	12144.00 (8440-12785)	11666.50 (8472.0-12499.0)	12357.5 (11580-13170)	11536 (11411-12630)	N/A	N/A	N/A	N/A	N/A
IL-3	8.37 (3.14-19.80) **	11.61 (0.00-26.11) **	0.00 (0.00-1.18)	0.00 (0.00-0.89)	9.43 (1.85-17.10) §	13.98 (5.66-29.85) §	8.45 (5.28-19.32)¤	17.07 (7.71-1.27)¤	-0.09/ 0.81	0.87/ $4x10^{-3}$	0.58/ 0.12	0.25/ 0.54	0.09/ 0.81
IL-4	23.82 (21.46-233.71) **	17.42 (8.29-147.57)	9.04 (7.94-9.63)	8.69 (8.29-9.47)	22.31 (16.46-116.27) §	22.30 (15.36-68.74) §	25.97 (19.47-156.36) ¤	14.74 (13.53-114.94)¤	0.43/ 0.27	0.53/ 0.17	-0.14/ 0.72	0.50/ 0.20	-0.43/ 0.27
IL-5	263.05 (138.37-4492.00) **	479.98 (0.38-5173) **	0.43 (0.38-0.48)	0.43 (0.38-0.48)	229.61 (139.6-2252)§	461.92 (296.37-2914.00) §	441.27 (177.61-3178) ¤	565.33 (288.07-4066)¤	0.68/ 0.06	0.09/ 0.81	-0.68/ 0.06	0.75/ 0.03	-0.68/ 0.06
IL-6	4.55 (0.85-12.18) **	14.31 (4.30-4506)	1342 (1234-3718)	1682.00 (1465-4506)	9001 (6223-10981) *§€	10774.50 (9702.0-11183) *§€	3729.00 (1595-9405)#¤	8779.5 (4605-10574) #¤	-0.53/ 0.17	0.53/ 0.17	0.78/ 0.02	0.12/ 0.76	0.53/ 0.17

IL-7	0.50 (0.42-0.72) **	0.59 (0.46-0.84)	0.32 (0.25-0.57)	0.39 (0.28-0.84)	1.21 (0.64-2.33) *§	2.05 (1.62-5.32) *§€	0.77 (0.51-1.62)#¤	1.24 (1.00-2.31)#¤	0.07/ 0.86	0.31/ 0.44	0.04/ 0.90	0.44/ 0.27	-0.07/ 0.86
IL-8	16.83 (11.74-42.19) **	29.12 (15.50-130.52) **	228.35 (67.31-405.28)	337.58 (80.89-591.61)	4915.50 (2068-6856) *§€	7139.00 (3934-7923) *§€	2250.50 (841.84-5648)#¤	4927 (1555-6890)#¤	0.53/ 0.17	0.24/ 0.56	-0.43/ 0.27	0.62/ 0.09	-0.53/ 0.17
IL-9	51.89 (44.79-174.95) **	56.28 (37.89-189.85) **	37.72 (36.32-38.52)	37.93 (35.51-38.39)	55.29 (47.74-152.1)§	66.55 (53.18-171.23) §	60.38 (51.66-129.15) ¤	71.23 (50.57-150.12)¤	0.24/ 0.56	0.73/ 0.03	0.09/ 0.81	0.75/ 0.03	-0.24/ 0.56
IL-10	331.08 (60.55-1460.0) **	333.67 (0.52-1633.0) **	0.36 (0.210-0.45)	0.39 (0.31-0.52)	484.51 (72.48-1189)§	485.09 (90.43-1383)§	390.79 (121.6-995.76) ¤	328.1 (117.12-1231)	0.34/ 0.40	0.82/ 0.01	0.24/ 0.56	-0.12/ 0.76	-0.34/ 0.40
IL-12p40	0.74 (0.61-0.88) **	0.99 (0.74-1.46)	1.06 (0.81-1.31)	1.09 (0.95-1.46)	2.60 (2.07-3.22) *§	4.02 (3.30-9.41) *§€	2.15 (1.38-2.54)#¤	2.70 (2.54-4.30)#¤	0.22/ 0.59	0.46/ 0.24	0.00/ 1.00	0.51/ 0.19	-0.22/ 0.59
IL-12p70	0.59 (0.50-0.84)	0.58 (0.46-0.73)	0.50 (0.42-0.57)	0.48 (0.42-0.53)	1.44 (1.03-4.48) *§€	2.91 (1.09-10.65) *§€	0.74 (0.57-1.07)¤	1.00 (0.69-1.38)#¤	0.09/ 0.81	0.29/ 0.48	0.00/ 1.00	0.50/ 0.20	-0.09/ 0.81
IL-13	20.51 (16.91-147.07) **	29.75 (0.68-178.86) **	0.64 (0.60-0.78)	0.68 (0.60-0.80)	14.51 (10.78-82.4) *§€	18.01 (13.23-89.54) §	19.40 (16.58-95.20)¤	22.17 (16.86-120.01)¤	-0.48/ 0.21	0.09/ 0.81	0.43/ 0.27	0.25/ 0.54	0.48/ 0.21
IL-15	0.48 (0.41-0.57) **	0.57 (0.46-0.65) **	0.41 (0.36-0.47)	0.45 (0.410-0.49)	1.47 (1.29-3.07)	2.42 (1.85-	0.67 (0.57-1.76)#¤	1.30 (0.77-2.30)#¤	0.09/ 0.81	0.29/ 0.48	0.00/ 1.00	0.50/ 0.20	-0.09/ 0.81

					*§€	5.34) *§€								
IL-17A	8.77 (1.91-60.33) **	7.66 (1.58-134.62) **	1.15 (0.88-1.58)	1.32 (0.62-1.58)	23.89 (4.51-70.87) §	71.73 (9.07-168.41) §	24.24 (4.27-86.20)¤	74.61 (4.39-156.33)¤	0.39/ 0.33	0.78/ 0.02	0.00/ 1.00	0.75/ 0.03	-0.39/ 0.33
IL-17E/ IL-25	0.17 (0.00-0.37)	0.37 (0.00-0.57) **	0.00	0.00	0.78 (0.58-1.06)* §€	1.43 (0.76-2.38) *§	0.50 (0.40-0.99)#¤	0.96 (0.42-1.80)#¤	-0.02/ 0.95	0.71/ 0.04	0.34/ 0.40	0.63/ 0.09	0.02/ 0.95
IL-17F	0.00	0.00	0.00	0.00	0.00	0.00 (0.0-0.59)	0.00	0.00	-0.50/ 0.19	0.16/ 0.68	0.50/ 0.19	0.21/ 0.60	0.50/ 0.19
IL-18	0.49 (0.44-0.64)	0.67 (0.54-0.74) **	0.22 (0.00-0.54)	0.47 (0.44-0.54)	0.98 (0.70-1.67)* §	1.73 (0.91-5.43) *§€	0.80 (0.47-0.93)#¤	1.20 (0.86-1.25)#¤	0.19/ 0.64	0.19/ 0.64	-0.19/ 0.64	0.75/ 0.03	-0.19/ 0.64
IL-22	0.76 (0.66-0.93)	0.80 (0.77-1.17)	0.89 (0.71-1.14)	1.04 (0.68-1.17)	1.91 (1.55-2.64)* §	3.09 (2.14-4.42) *§€	1.43 (1.03-2.26)#¤	2.09 (1.36-2.66)#¤	0.00/ 1.00	0.78/ 0.02	0.39/ 0.33	0.50/ 0.20	0.00/ 1.00
IL-27	4.68 (4.47-4.88)	4.78 (4.32-5.27)	4.78 (4.47-4.88)	4.61 (4.10-5.01)	6.61 (5.75-8.10)* §€	8.31 (6.31-11.48) *§	5.54 (5.39-6.15)#¤	6.70 (5.63-7.49)#¤	0.19/ 0.64	0.19/ 0.64	-0.19/ 0.64	0.75/ 0.03	-0.19/ 0.64
IP-10	0.94 (0.63-1.35) **	1.14 (0.68-2.27) **	0.55 (0.45-0.86)	0.57 (0.49-0.81)	1067.00 (982.47-1220.0) * §€	1317.00 (1058-1518) §*€	60.78 (28.01-920.58) #¤	295.81 (79.18-1021)#¤	0.00/ 1.00	0.0/ 1.00	0.00/ 1.00	0.00/ 1.00	0.00/ 1.00

MCP-1	0.87 (0.76-1.15) **	1.31 (0.89-186.11)	33.69 (12.76-102)	91.29 (44.72-186.11)	1941 (1624-2114) *§€	2120.5 (2024-2165) *§€	677.03 (304-1478)#¤	1962 (1639-2127)#¤	-0.19/ 0.64	0.19/ 0.64	0.29/ 0.48	0.00/ 1.00	0.19/ 0.64
MCP-3	1.65 (1.39-1.82)	1.87 (1.71-5.74)	1.76 (1.45-4.82)	2.59 (1.66-5.74)	78.40 (46.52-228.11) *§€	448.74 (108.42 - 795.75) *§€	13.65 (4.68-54.79)#¤	84.45 (13.12-138.26) #¤	0.29/ 0.48	0.48/ 0.21	-0.04/ 0.90	0.50/ 0.20	-0.29/ 0.48
M-CSF	3.35 (2.68-10.13) **	7.61 (2.83-19.87) **	1.35 (0.78-1.90)	2.56 (1.41-3.18)	10.47 (7.05-17.14)* §	20.31 (16.24-26.38) *§	8.43 (6.98-13.60)¤	18.50 (13.31-22.71)¤	0.19/ 0.64	0.78/ 0.02	0.19/ 0.64	0.62/ 0.09	-0.19/ 0.64
MDC/ CCL22	3.08 (1.64-6.53) **	9.36 (0.83-23.45) **	0.70 (0.0-1.04)	0.80 (0.00-1.20)	7.18 (3.63-20.63)* §€	26.52 (11.98-68.67) *§€	1.61 (1.30-3.68)¤	3.72 (1.68-13.73)¤	-0.19/ 0.64	0.00/ 1.00	0.09/ 0.81	0.37/ 0.35	0.19/ 0.64
MIG	8.75 (4.33-23.11) **	22.86 (1.68-53.98) **	1.41 (1.28-1.74)	1.47 (1.41-1.68)	500.40 (306.69 - 1699.0) * §€	4705.5 0 (3640-5050) *§€	18.78 (11.94-353.56)¤	92.32 (34.77-1583)#¤	-0.29/ 0.48	0.09/ 0.81	0.29/ 0.48	0.12/ 0.76	0.29/ 0.48
MIP-1α	53.47 (18.36-148.81) **	168.19 (5.33-565.77) **	2.22 (1.97-3.76)	2.40 (2.24-5.33)	63.47 (15.08-107.76) §	271.68 (89.21-341.66) §	54.96 (32.85-110.54)¤	197.03 (111.89-411.17)¤	0.58/ 0.12	0.58/ 0.12	-0.29/ 0.48	0.75/ 0.03	-0.58/ 0.12
MIP-1β	116.10 (58.01-427.83) **	372.28 (14.63-2069)**	3.54 (3.32-14.38)	3.77 (3.10-14.63)	109.59 (34.57-201.30) §	364.65 (180.84 - 602.38) §	123.54 (89.97-287.54)¤	463.10 (179.66-1260)¤	0.68/ 0.06	0.48/ 0.21	-0.43/ 0.27	0.75/ 0.03	-0.68/ 0.06

PDGF-AA	1.58 (0.85-1.85)	3.13 (1.39-4.25)	1.65 (1.12-6.74)	1.37 (1.20-6.11)	2.17 (1.61-6.21)*	2.07 (1.61-6.38)	2.26 (1.79-5.47)#	3.41 (1.65-8.49)	-0.34/ 0.40	0.14/ 0.72	0.24/ 0.56	0.75/ 0.03	0.34/ 0.40
PDGF-AB/BB	1.55 (1.37-2.69)	2.21 (1.61-2.63)	1.97 (1.55-3.05)	2.03 (1.61-2.81)	4.08 (2.45-8.23)* §	5.20 (4.85-9.5) *§€	2.90 (1.97-6.40)#	3.45 (2.33-4.96) # ¤	-0.39/ 0.33	0.00/ 1.00	0.34/ 0.40	0.00/ 1.00	0.39/ 0.33
RANTES	103.83 (69.40-246.02) **	564.80 (13.13-1446) **	1.28 (0.40-5.73)	3.02 (0.63-13.13)	194.79 (174.65 - 597.86) §€	15550 (1022-1888) *§€	110.98 (61.77-154.20)¤	798.21 (189.85-1103.00) ¤	0.04/ 0.90	0.34/ 0.40	0.04/ 0.90	0.62/ 0.09	-0.04/ 0.90
TGFα	0.00 (0.00-0.60) **	0.56 (0.42-0.66) **	0.99 (0.54-2.22)	1.08 (0.58-1.48)	3.01 (2.02-5.30)* §€	4.21 (2.24-5.92) *§	1.36 (1.15-3.39)#	2.53 (1.36-3.63) # ¤	0.19/ 0.64	0.19/ 0.64	-0.19/ 0.64	0.75/ 0.03	-0.19/ 0.64
TNFα	20.78 (7.29-26.63) **	27.16 (3.08-39.70) **	2.10 (1.92-3.30)	2.21 (2.07-3.08)	21.38 (13.44-28.11) §	27.48 (15.69-35.11) §	28.27 (13.00-44.61)¤	32.52 (12.04-53.09) ¤	-0.19/ 0.64	-0.19/ 0.64	-0.09/ 0.81	0.75/ 0.03	0.19/ 0.64
TNFβ	17.18 (5.86-43.42) **	9.79 (2.56-51.27)	5.06 (4.37-8.32)	6.18 (5.00-8.05)	20.93 (18.21-40.05) §	22.96 (16.66-41.21) §	24.57 (11.20-63.71)¤	21.45 (10.88-76.26) ¤	-0.48/ 0.21	-0.09/ 0.81	0.24/ 0.56	0.62/ 0.09	0.48/ 0.21
VEGF-A	0.95 (0.79-2.79) **	1.14 (0.84-41.35) **	25.63 (14.83-36.78)	32.98 (21.05-62.53)	27.98 (13.39-41.43)* €	95.58 (42.25-175.53) *§€	7.00 (2.93-24.58)#¤	26.00 (10.48-53.11) #	-0.29/ 0.48	0.48/ 0.21	0.53/ 0.17	0.12/ 0.76	0.29/ 0.48

Supplementary Table 3 Correlations between cytokine levels in the cocultures and Treg proliferation. The table lists correlations between proliferation of Tregs from direct and indirect cocultures and levels of cytokines in their culture supernatants (n=12). Concentrations of all listed mediators were significantly higher in direct, than in indirect cocultures. The data were analyzed with Spearman's rank correlation, R and p values are given for each cytokine.

Cytokine	R	p
MCP-3	-0.9	$4x10^{-5}$
IL-22	-0.9	$4x10^{-5}$
IL-12p70	-0.85	$4x10^{-4}$
EGF	-0.83	$1x10^{-3}$
Eotaxin	-0.82	$1x10^{-3}$
IL-12p40	-0.81	$1x10^{-3}$
IL-6	-0.80	$1x10^{-3}$
MIG	-0.79	$1x10^{-3}$
IL-1β	-0.79	$1x10^{-3}$
IL-15	-0.79	$2x10^{-3}$
IL-7	-0.78	$2x10^{-3}$
IP-10	-0.76	$3x10^{-3}$
VEGF-A	-0.75	$4x10^{-3}$
IL-8	-0.74	$5x10^{-3}$
IL-18	-0.72	$7x10^{-3}$
RANTES	-0.72	$7x10^{-3}$
Fractalkine	-0.67	0.01
MCP-1	-0.64	0.02
MDC/CCL22	-0.64	0.02
FLT-3L	-0.48	0.1
PDGF-AB/BB	-0.41	0.17
