# Supplementary material for: Mesenchymal stem cells transfer mitochondria to allogeneic Tregs in an HLA-dependent manner improving their immunosuppressive activity
Source: Nat Commun. 2022 Feb 14;13:856. doi: 10.1038/s41467-022-28338-0 (PMC8844425; doi:10.1038/s41467-022-28338-0)
Supplement: Supplementary file 3 — Description of Additional Supplementary Files [file 41467_2022_28338_MOESM3_ESM.pdf]

Description of Additional Supplementary Files

Supplementary Movie 1. Direct Treg- ASC coculture. The movie contains fragments from 3 independent Treg-ASC cocultures. Photos were captured with JuLi microscope (NanoEntek) with 2-minute intervals with 4x objective lenses.
