# Supplementary material for: Mesenchymal stem cells transfer mitochondria to allogeneic Tregs in an HLA-dependent manner improving their immunosuppressive activity
Source: Nat Commun. 2022 Feb 14;13:856. doi: 10.1038/s41467-022-28338-0 (PMC8844425; doi:10.1038/s41467-022-28338-0)
Supplement: Supplementary file 5 — Reporting summary [file 41467_2022_28338_MOESM5_ESM.pdf]

nature portfolio

Corresponding author(s): Natalia Marek-Trzonkowska

Last updated by author(s): Dec 18, 2021

Reporting Summary

Nature Portfolio wishes to improve the reproducibility of the work that we publish. This form provides structure for consistency and transparency in reporting. For further information on Nature Portfolio policies, see our Editorial Policies and the Editorial Policy Checklist.

Statistics

For all statistical analyses, confirm that the following items are present in the figure legend, table legend, main text, or Methods section.

n/a	Confirmed	
☐	☒	The exact sample size (n) for each experimental group/condition, given as a discrete number and unit of measurement
☐	☒	A statement on whether measurements were taken from distinct samples or whether the same sample was measured repeatedly
☐	☒	The statistical test(s) used AND whether they are one- or two-sided<br>*Only common tests should be described solely by name; describe more complex techniques in the Methods section.*
☒	☐	A description of all covariates tested
☐	☒	A description of any assumptions or corrections, such as tests of normality and adjustment for multiple comparisons
☐	☒	A full description of the statistical parameters including central tendency (e.g. means) or other basic estimates (e.g. regression coefficient) AND variation (e.g. standard deviation) or associated estimates of uncertainty (e.g. confidence intervals)
☐	☒	For null hypothesis testing, the test statistic (e.g. F, t, r) with confidence intervals, effect sizes, degrees of freedom and P value noted<br>*Give P values as exact values whenever suitable.*
☒	☐	For Bayesian analysis, information on the choice of priors and Markov chain Monte Carlo settings
☒	☐	For hierarchical and complex designs, identification of the appropriate level for tests and full reporting of outcomes
☐	☒	Estimates of effect sizes (e.g. Cohen's d, Pearson's r), indicating how they were calculated

Our web collection on statistics for biologists contains articles on many of the points above.

Software and code

Policy information about availability of computer code

Data collection	BD FACS Diva v8.01 software; Thermo Scientific Xcalibur Software (v.2.0, ThermoScientific)
Data analysis	Statistica 13.0 software (Statsoft, Poland); BD FACS Diva v8.01 software; FlowJo v7.6 software; HLAMatchmaker V3.1 alogorithm available at http://www.epitopes.net/downloads.html; ClustVis 2.0 tool available online at http://biit.cs.ut.ee/clustvis/#mathematics; MiaFora NGS software

For manuscripts utilizing custom algorithms or software that are central to the research but not yet described in published literature, software must be made available to editors and reviewers. We strongly encourage code deposition in a community repository (e.g. GitHub). See the Nature Portfolio guidelines for submitting code & software for further information.

Data

Policy information about availability of data

All manuscripts must include a data availability statement. This statement should provide the following information, where applicable:

- Accession codes, unique identifiers, or web links for publicly available datasets
- A description of any restrictions on data availability
- For clinical datasets or third party data, please ensure that the statement adheres to our policy

All data needed to evaluate the conclusions in the paper are present in the main manuscript, the Supplementary Materials and the Source data. Source data are provided with this paper and comprise the HLA sequencing raw data in XML format and a Source Data.xlsx file that presents all the relevant raw data from each figure in the main manuscript and in the Supplementary Materials. The remaining raw data are available upon request from the corresponding author (N.M.T.) after signing confidentiality agreement because of the patent pending. The requests should be directed to the corresponding author to the e-mail address natalia.marek-trzonkowska@ug.edu.pl.

Field-specific reporting

Please select the one below that is the best fit for your research. If you are not sure, read the appropriate sections before making your selection.

- [x] Life sciences
- [ ] Behavioural & social sciences
- [ ] Ecological, evolutionary & environmental sciences

For a reference copy of the document with all sections, see nature.com/documents/nr-reporting-summary-flat.pdf

Life sciences study design

All studies must disclose on these points even when the disclosure is negative.

Sample size	The minimal sample size necessary to achieve reliable measurements was adjusted individually for each type of analysis based on existing literature and our previous experience. (e.g. PMID: 32778404 or PMID: 34750385). Sample size was determined to be adequate based on the magnitude and consistency of measurable differences between groups. At least 4 times of independent experiments would be considered significant for statistical analysis.
Data exclusions	No outliers were excluded.
Replication	In aim to verify the reproducibility of experimental findings we performed the cocultures and organelle transfer experiments with cells collected from a group of healthy donors of both genders. Thus, the experiments were performed independently with cells derived from different donors to confirm the experimental findings. Number of each experiment is given in the manuscript. Experiments with blocking anti-HLA class II antibodies were performed in duplicates.
Randomization	Sample randomization was not relevant to this study as each sample was tested simultaneously in various experimental settings.
Blinding	Blinding of the donor samples was not relevant to this study. All donors recruited were healthy individuals and the study did not aim to test the differences between various groups of the patients. In addition, HLA typing was performed after flow cytometry analyses. Thus, the HLA eplet mismatch loads were unknown during organelle transfer experiments.<br><br>Blinding samples of different cell types was not relevant to this study. Blinding Treg and ASC, as well as Treg and K562 cell samples would have precluded organelle transfer experiments. For these experiments the researcher had to identify the cell type to stain ASCs and K562 cells in aim to track studied cellular elements. It was mandatory to keep Tregs unlabelled for these experiments. Even if the cell staining and cell analysis would be performed by two independent researchers, blinding of the cell types for analysis was not possible. Tregs and ASCs, as well as Tregs and K562 cells differ significantly in morphology, size and surface antigen expression. Thus, they can be easily discriminated during flow cytometry data collection with different SSC and FSC values regardless any blinding or lack of specific antibody labelling.

Reporting for specific materials, systems and methods

We require information from authors about some types of materials, experimental systems and methods used in many studies. Here, indicate whether each material, system or method listed is relevant to your study. If you are not sure if a list item applies to your research, read the appropriate section before selecting a response.

Materials & experimental systems

n/a	Involved in the study	
[ ]	[x]	Antibodies
[ ]	[x]	Eukaryotic cell lines
[x]	[ ]	Palaeontology and archaeology
[x]	[ ]	Animals and other organisms
[ ]	[x]	Human research participants
[x]	[ ]	Clinical data
[x]	[ ]	Dual use research of concern

Methods

n/a	Involved in the study	
[x]	[ ]	ChIP-seq
[ ]	[x]	Flow cytometry
[x]	[ ]	MRI-based neuroimaging

Antibodies

Antibodies used	If not otherwise stated the antibodies were used in amount of 5µl/test in 1:20 dilution:<br>Anti-human CD3 PacificBlue, clone UCHT1; cat. no. 558117 (BD Biosciences, USA)<br>Anti-human CD4 APC, clone RPA-T4 , cat.no. 561841 (BD Biosciences, USA)<br>Anti-human CD4 PerCP, clone SK3 (also known as Leu3a); cat no. 340671 (BD Biosciences, USA), this antibody was used in an amount of 10µl/test in 1:10 dilution<br>Anti-human CD4 V500, clone RPA-T4; cat.no. 560768 (BD Biosciences, USA)<br>Anti-human CD25 PE, klon M-A251; cat. no. 555432 (BD Biosciences, USA)<br>Anti-human CD127 FITC, clone hIL-7R-M21; cat. no. 560549 (BD Biosciences, USA)

Anti-human CD8 PerCP, clone SK1; cat. no. 347314 (BD Biosciences, USA)
Anti-human CD16PerCP-Cy5.5, clone 3G8, cat. no. 338440 (BD Biosciences, USA)
Anti-huma CD19 PerCP, clone 4G7; cat no. 340865 (BD Biosciences, USA)
Anti-huma CD14 PerCP, clone MϕP9; cat no. 340660 (BD Biosciences, USA)
Anti-human CD45RA PE-Cy7, clone L48; cat. no. 337186 (BD Biosciences, USA)
Anti-human CD62L APC-Cy7, clone DREG-56; cat. no. 304814 (BioLegend, USA)
Anti-human CD304 (Neuropilin-1) PE-Cy7, clone 12C2; cat no. 354508 (BioLegend, USA)
Anti-human Foxp3 APC; clone: 236A/E7; cat. no. 17-4777-42 (eBiosciences, USA)
Anti-human Foxp3 FITC, clone PCH101, cat. no. 11-4776-42 (eBiosciences, USA)
Anti-Human CD73 APC, clone AD2; cat.no. 17-0739-42 (eBiosciences, USA)
Anti-human CD152 (CTLA-4) PE, clone 14D3; cat. no. 12-1529-42 (eBiosciences, USA)
Anti-human CD39 PE-Cy7,clone eBioA1 (A1); cat. no. 25-0399-42 (eBiosciences, USA)
Anti-human CD31 APC-Cy7, clone WM59; cat no. 303120 (BioLegend, USA)
Anti-human Helios eFluor® 450, clone 22F6; cat no. 48-9883-42 (eBiosciences, USA)
Anti-human CD105 APC-eFluor780, clone SN6; cat. no. 47-1057-42 (eBiosciences, USA)
Anti-human HLA-ABC- Alexa Fluor 700, clone W6/32; cat no. 56-9983-42 (Invitrogen, USA)
Anti-Human HLA-DR APC, clone LN3; cat. no. 17-9956-42 (eBiosciences, USA)
Anti-human CD69 BV650, clone FN50; cat no. 563835 (BD Biosciences, USA)
Anti-human HSP-60 PE, clone LK-2; cat no. ab82518 (Abcam, UK), this antibody was used in an amount of 1µl/test in 1:100 dilution
Anti-human HSP-70 PE, clone N27F34; cat no. ab65174 (Abcam, UK),this antibody was used in an amount of 1µl/test in 1:100 dilution
Anti-human HSP-90 PE, clone AC88; cat no. ab65171 (Abcam,UK), this antibody was used in an amount of 1µl/test in 1:100 dilution
Anti-human CD90 PE, clone eBio5E10(5E10); cat.no. 12-0909-42 (eBiosciences, USA)
Anti-human CD44 FITC, clone G44-26 (also known as CD26); cat no. 555478 (BD Biosciences , USA)
Anti-human CD45 V500, clone HI30; cat. no. 560777 (BD Biosciences, USA)
Anti-human HLA-DR PerCP, clone L243; cat.no. 347402 (BD Biosciences, USA), this antibody was used in an amount of 10µl/test in 1:10 dilution
Anti-human HLA class II antibody unconjugated; clone IVA12, cat. no. CBT-104701 (BIOZOL Diagnostica Vertrieb GmbH, Germany), this antibody was used in concentration 10µg/ml

Validation

All conjugated primary antibodies used in the study were validated by the manufacturers for identification of human antigens and flow cytometry application, as described on the manufacturers' websites.

The primary unconjugated antibody used in the study was validated by the manufacturer to bind the shared epitopes of human leucocyte antigen (HLA) class II molecules HLA-DP, HLA-DQ and HLA-DR. It was also validated with the previous studies as anti-human HLA class II blocking antibody (e.g. paper PMID: 21294723).

Eukaryotic cell lines

Policy information about cell lines

Cell line source(s)	Human erythromyeloblastoid K562 cell line
Authentication	Morphology analysis with microscopy and confirmation of HLA-ABC- HLA-DR- phenotype with flow cytometry was used for cell line authentication.
Mycoplasma contamination	All cell lines were tested negative for mycoplasma contamination.
Commonly misidentified lines (See ICLAC register)	No misidentified cell lines were used in the study.

Human research participants

Policy information about studies involving human research participants

Population characteristics	Donors of Tregs- female and male healthy volunteer blood donors, no history of chronic disease, tested negative for HBV, HCV and HIV, 40-60 years old.<br><br>Donors of ASCs- female and male donors admitted for liposuction procedure, no history of chronic disease, tested negative for HBV, HCV and HIV, 40-60 years old.
Recruitment	Only healthy individuals were recruited to exclude the impact of any drug treatment or chronic condition on the immune status of the patients. All patient samples were tested negative for HCV, HBV and HIV. Patients of both genders were recruited to avoid misleading conclusions that could refer only to one gender. In each experiment male and female cells were tested and the male and female group sizes were equal. Patients at age of 40-60 years old were recruited to avoid age related deviation in ASC and Treg phenotype and function. However, the adipose tissue donors had higher BMI as compared with the T cell donors. The patients were recruited according to the objective criteria of age, gender and health status and no self-selection bias affected the recruitment.
Ethics oversight	All blood and adipose tissue donors gave informed consent and all experimental protocols were approved by Independent Bioethics Commission for Research of the Medical University of Gdańsk- agreement no. NKEBN/353/2011.

Note that full information on the approval of the study protocol must also be provided in the manuscript.

Flow Cytometry

Plots

Confirm that:

- [x] The axis labels state the marker and fluorochrome used (e.g. CD4-FITC).
- [x] The axis scales are clearly visible. Include numbers along axes only for bottom left plot of group (a 'group' is an analysis of identical markers).
- [x] All plots are contour plots with outliers or pseudocolor plots.
- [x] A numerical value for number of cells or percentage (with statistics) is provided.

Methodology

Sample preparation	Tregs and CD4+ Tconvs were isolated from buffy coats derived from female and male healthy donors. Buffy coats were first diluted in 1:1 proportion with PBS and peripheral blood mononuclear cells (PBMC) were obtained by Ficoll/Uropoline gradient centrifugation. Then, CD4+ T cells were isolated with negative immunomagnetic selection method with EasySep Human CD4+ T Cell Enrichment Kit (Stemcell Technologies; selection purity 90–99%). Subsequently, CD4+ T cells were labelled with monoclonal antibodies (BD Biosciences, USA) specific for CD3, CD4, CD25, CD127, CD8, CD19, CD16 and CD14 antigens. The last 4 mAbs were conjugated with the same fluorochrome in aim to cut-off in one step cytotoxic T cells (Tc), B cells, NK cells and monocytes, respectively. These cells were defined all together in sorting algorithm as lineage. Then, the cells were sorted with florescence activated cell sorter (FACS; Aria II, BD Biosciences) into the following phenotype of Tregs: CD3+CD4+CD25High CD127−/LowDoublet−Lineage− and Tconvs: CD3+CD4+CD25-CD127HighDoublet−Lineage−. The post-sort purity of Tregs and Tconvs was ~100% [median(min-max): 98%(97-99)].<br><br>ASCs were isolated from fresh samples of adipose tissue (50-200 ml) obtained during liposuction procedures from healthy individuals. The samples were triple washed with PBS to remove debris and erythrocytes. Subsequently, digestion solution (DS) was added in 1:1 of DS (ml): tissue mass (g) ratio at 37°C with gentle agitation to obtain single cell suspension. Composition of DS solution was as follows: 5% collagenase type I (Sigma-Aldrich; 5mg/ml), 19% of PBS and 1% of foetal bovine serum (FBS). The collagenase was inactivated with an equal volume of 10% FBS supplemented LG-DMEM medium (PAA), followed by filtration of the resulting cell suspension through a 100 micrometer nylon cell strainer (Falcon). The filtrate was centrifuged and washed with PBS. Then, the pellet was suspended in erythrocyte lysis buffer for 10 min. (at room temperature, RT) to remove erythrocytes. Subsequently, the cells were centrifuged (600g, 10 min.) and washed with 4% FBS supplemented PBS. Isolated cells represented an initial stromal vascular cell fraction (SVF). Following the first 5 days of initial plating, nonadherent cells were removed by intense washing and the remaining fibroblast-like adherent cells were maintained.
Instrument	Instrument used for Treg sorting- FACS Aria II, BD Biosciences.<br><br>Instrument used for flow cytometry analysis of cells- LSRFortessa, BD Biosciences.
Software	BD FACS Diva v8.01 software
Cell population abundance	The post-sort purity of Tregs and Tconvs was ~100% [median(min-max): 98%(97-99)]. The post-sort purity was determined with flow cytometry (LSRFortessa, BD Biosciences). For post-sort purity evaluation Tregs were identified as CD4+CD25High CD127Low/-FoxP3+ cells, while Tconvs were identified as CD4+CD25-CD127HighFoxP3- cells.

Gating strategy	Gating strategy is presented and described in details in Fig.6, Fig. S2 and Fig. S3. Briefly, Tregs and ASCs were identified as SSCLowCD105- and SSCHighCD105+ cells, respectively.<br><br>K562 cells and Tregs were identified as HLA-ABC-SSCHigh and Tregs HLA-ABC+SSCLow cells, respectively.<br><br>Proliferation index of Tconvs was calculated by using the number of cells measured in each division peak. For this purpose live Tconvs were identified as 7-ADD- and VPD-450+ cells. The division peaks in 7-ADD-VPD-450+ population were numbered and the numbers of cells measured in each division peak were used for calculation of proliferation index.

☒ Tick this box to confirm that a figure exemplifying the gating strategy is provided in the Supplementary Information.
